# Balancing the scales: Preliminary investigation of total energy expenditure and daily metabolizable energy intake in Matschie’s tree kangaroo (*Dendrolagus matschiei*)

**DOI:** 10.1371/journal.pone.0270570

**Published:** 2022-06-27

**Authors:** Noah T. Dunham, Diana C. Koester, Ellen S. Dierenfeld, Rebecca Rimbach, Herman Pontzer

**Affiliations:** 1 Division of Conservation and Science, Cleveland Metroparks Zoo, Cleveland, OH, United States of America; 2 Department of Biology, Case Western Reserve University, Cleveland, OH, United States of America; 3 Ellen S. Dierenfeld, LLC, St. Louis, MO, United States of America; 4 Nottingham Trent University, Southwell, United Kingdom; 5 Evolutionary Anthropology, Duke University, Durham, NC, United States of America; 6 Duke Global Health Institute, Duke University, Durham, NC, United States of America; Universidad de Guadalajara, MEXICO

## Abstract

Matschie’s tree kangaroo (*Dendrolagus matschiei*) is an endangered arboreal marsupial native to Papua New Guinea. Detailed field studies of its behavior and ecology are scarce due largely to its occupation of remote cloud forests and cryptic nature. Although this species has been in human care since the 1950s, much of its biology is still unknown. The current *ex situ* population is not sustainable due to health and reproductive problems, believed to stem largely from issues with diet and obesity. To better assess potential discrepancies between energy requirements and energy intake, we sought to 1) quantify total energy expenditure (TEE) of two zoo-housed Matschie’s tree kangaroos (body mass = 9.0–9.7 kg) on a diet composed largely of leafy browse; 2) quantify food and macronutrient intake, apparent dry matter macronutrient digestibility, and metabolizable energy (ME) intake over a 14-month period; and 3) test for seasonal changes in ME intake due to seasonal differences in the varieties of leafy browse offered. Using the doubly labeled water method, we determined TEE for the female (288 kcal day ^-1^) and male (411 kcal day ^-1^). Resulting mean TEE was well below the expected value for marsupials and macropods (i.e., ~60% of the expected value based on body mass). The mean calculated ME intakes for the female and male were 307 kcal day^-1^ and 454 kcal day^-1^, respectively. There were significant seasonal differences in ME intakes, driven by reduced intake in the autumn. These results demonstrate that Matschie’s tree kangaroos can be maintained at healthy body weights and conditions on fiber-rich and browse-heavy diets. Our findings contribute important insights into tree kangaroo energetics and physiology and can be applied to help reformulate the diet of Matschie’s tree kangaroos at captive facilities to improve population health and sustainability.

## Introduction

Matschie’s tree kangaroo (also known as the Huon tree kangaroo; *Dendrolagus matschiei*) is an arboreal marsupial native only to the Huon peninsula of Papua New Guinea with an estimated ~2,500 adult individuals remaining [1]. Detailed field studies of the behavior and diet of this species are scarce due in part to its dwindling population size and because of its cryptic nature in remote cloud forests [2]. Matschie’s tree kangaroos are currently housed at 18 Association of Zoos and Aquariums (AZA) accredited zoological institutions; however, the captive population is unsustainable due to various reproductive and health issues which are likely linked to problems with diet and obesity [3–5].

While little is known about the feeding ecology of Matschie’s tree kangaroo, Dierenfeld et al. [6] recently quantified the macronutrient composition of foods consumed by free-ranging *Dendrolagus matschiei* (i.e., n = 24 plant items, including leaves, stems, and petioles) and found that food items averaged moderate crude protein (11±5%, dry matter basis; DMB) and nonstructural carbohydrate (27±8%) content, low starch (1±1%) and crude fat (3±2%) content, and high neutral detergent fiber (52±13%) content. Free-ranging tree kangaroos likely receive a substantial portion of their metabolizable energy from fiber via fermentation in the foregut. However, there are no published accounts regarding the degree of fiber and macronutrient digestibility in any tree kangaroo species.

The macronutrient composition of the wild diet is in strong contrast with the diets of zoo-housed individuals, whose diets typically contain a mixture of grain-based commercial biscuits/pellets, fruits, vegetables, and leafy greens which are considerably lower in fiber, richer in starch and soluble sugars, and more calorically dense than items consumed by free-ranging Matschie’s tree kangaroos [6, 7]. While local leafy browses (i.e., leafed woody branches) offered at some zoos better resemble the nutrient composition of wild diet items [8], browse is largely considered a dietary supplement or enrichment and is generally not offered in sufficient quantities to be considered a primary diet item.

Zoo-housed Matschie’s tree kangaroos are, on average, ~30–40% heavier than free-ranging conspecifics [2], and ~25% of the zoo population is considered overweight or obese (AZA Institutional Representatives pers. comm., 2022). Zoo-housed Matschie’s tree kangaroos’ propensity to obesity is likely attributable to discrepancies in the macronutrient content of wild vs. zoo diets but is also likely related to the amount of food offered in zoo settings (i.e., issues of both food quality and quantity). As marsupials, tree kangaroos have significantly lower basal metabolic rates compared to eutherian mammals of comparable body mass [9]. For instance, the mean basal metabolic rate of two Matschie’s tree kangaroos was found to be roughly half (i.e., 55%) the value for similarly sized eutherian mammals [10, 11]. Still, there are no published estimates of total energy expenditure (TEE; also known as field metabolic rate) for any tree kangaroo species. TEE provides a more complete estimate of an animal’s daily energy requirements by accounting for the energetic costs of daily activity and thermoregulation, in addition to basal metabolism [12]. Taken together, tree kangaroos’ presumed reduced energy requirements, combined with zoo diets characterized by calorically dense food items rich in sugar and starch, make them particularly susceptible to overfeeding, obesity, and potential downstream health and reproductive issues (i.e., overweight females are more likely to be acyclic and less likely to successfully breed and produce offspring).

The primary objective of this study was for the first time, to quantify TEE of two zoo-housed Matschie’s tree kangaroos and compare our findings to published TEE values of other marsupial taxa and members of the Macropodidae, in particular. We predicted that Matschie’s tree kangaroos would generally fit the regression lines for body mass vs. TEE within the overall marsupial dataset as well as within the Macropodidae subset. Our second objective was to quantify food and macronutrient intake, apparent dry matter macronutrient digestibility, and metabolizable energy (ME) intake in zoo-housed Matschie’s tree kangaroos over a 14-month period. We tested for seasonal changes in metabolizable energy intake due to major seasonal differences in the types (i.e., species) of dietary leafy browse offered to the tree kangaroos in this study. Because reduced browse variety has been associated with decreased food intake in some folivorous marsupials [13, 14], we predicted that spring and summer would correspond to increased ME intake due to greater availability of leafy browse varieties compared to autumn and winter months. Finally, assuming our study subjects were in a state of energy balance throughout the study period, we predicted that TEE would be roughly equivalent to ME intake. This study will improve our limited understanding of tree kangaroo biology and ultimately help improve the health and welfare of zoo-housed Matschie’s tree kangaroos by providing recommendations to better tailor zoo diets to physiological requirements.

## Materials and methods

### Ethics statement

The research protocol reported in this manuscript was approved by Cleveland Metroparks Zoo’s Scientific Research and Animal Care and Use Committee (protocol #CS2019-024) and by San Diego Zoo Global’s Institutional Animal Care and Use Committee (protocol #AZA10292019).

### Study subjects

We collected data on two Matschie’s tree kangaroos: an 11-year-old female (International Studbook #503) and a 14-year-old male (International Studbook #489) housed at Cleveland Metroparks Zoo in Cleveland, OH, USA. Prior to this study, the female’s body condition score was obese (i.e., 9/9) [15]; therefore, her diet was adjusted in a stepwise manner to promote weight loss primarily by increasing amounts of leafy browse offered and reducing non-browse diet items (i.e., biscuits, fruits, vegetables, and leafy greens). The female reached an ideal body condition score prior to the start of the study (i.e., 5/9) [15]. The diet offered to her for the duration of this study was designed to maintain an ideal body condition score. Historically, the male has maintained an ideal body condition score. The two individuals were housed separately but shared space briefly while shifting between adjacent housing areas for several minutes for two to eight days once every ~60 days for breeding purposes. If breeding interest was shown during shifting, the pair was kept together for one to two hours under observation and separated after breeding occurred. The female did not become pregnant, give birth, or lactate during the study period. Individuals were rotated daily between an indoor public-facing exhibit and an indoor holding area that is not visible to the public. Both spaces were maintained at approximately 24°C, with exposure to a natural photoperiod through skylights, and provided climbing structures and substrates to allow for terrestrial and arboreal locomotion. Offered daily diet contained a variety of vegetables (female: 40 g; male: 185 g), fruits (female: 20 g; male: 20 g), leafy greens (female: 150 g; male: 300 g), commercial dry food (female: 8 g; male: 50 g; Mazuri® Leaf-Eater Primate Diet, Biscuits, Purina Mills, LLC, St. Louis, MO, USA), and various leafy browse species approved for tree kangaroos. We offered ~1 kg of leafed woody branches to our tree kangaroos each day (i.e., female: 1147 g ± 405 g; male: 986 ± 387 g). Browse pieces were placed on elevated platforms throughout the exhibit and in off-exhibit holding areas. This resulted in ~500 g of leafy material offered each day (i.e., female: 575 g ± 294 g, n = 270 days; male: 461 ± 243 g, n = 256 days). Thus, leafy browse (i.e., not accounting for weight of wood) constituted ~73% of the total diet (i.e., weight as-fed) for the female and ~45% of the total diet for the male. Leafy browse was gathered locally (Cleveland, OH, USA) from June–October and was shipped from a commercial supplier in Florida (USA) from November–May. Diet amounts were quantified as described below. Water was available for ad libitum consumption. Medication for a chronic arthritis condition in the female was provided as needed. Individuals were trained to climb on to a platform scale (LVS-XL 700; precision = 0.1 kg; LW Measurements LLC, Rohnert Park, CA, USA) for body mass measurements. We were able to record body mass values approximately once every two weeks for the female and approximately once every two months for the male (Table 1).

**Table 1:** Total energy expenditure (TEE) of zoo-housed Matschie’s tree kangaroos (*Dendrolagus matschiei*).

Parameter	Female	Male
Age (years)^a^	11	14
Body Mass (kg)^b^	9.7 ± 0.3	9.0 ± 0.2
FQ	0.93	0.93
Body Fat (%)	21.3	12.8
FFM (kg)	8.0	8.0
TEE (kcal day^-1^)	288	411
(kcal day^-1^ BW^-1^)	30	46
(kcal day^-1^ MBW^-1^)	52	79
(kJ day^-1^)	1205	1720
(kJ day^-1^ BW^-1^)	124	191
(kJ day^-1^ MBW^-1^)	219	331

^a^ Age at the start of the study in June 2020

^b^ Mean body mass (± standard deviation) over the study period from June 2020 –July 2021; FQ = food quotient; FFM = fat free mass; TEE = total energy expenditure and is reported in multiple formats; BW = body weight in kg; MBW = metabolic body weight in kg and was calculated using the formula:: MBW = BW^0.75^.

### Total energy expenditure (TEE)

We used the doubly labeled water (DLW) method [16, 17] to determine TEE. Both individuals ingested premeasured doses (female: 20.9 g, male: 18.1 g) of doubly labeled water (6% ^2^H_2_O, 10% H_2_^18^O) tailored to their body mass to provide sufficient initial isotopic enrichment [17]. Water doses were poured over and absorbed into dry food biscuits to ensure ingestion of the entire DLW dose. Due to challenges associated with the COVID-19 pandemic and training the animals for voluntary blood draws, samples were collected in November 2020 for the female and May 2021 for the male. We collected one blood sample (2 ml) prior to dosing, and another two (female) or three (male) blood samples post-dose ingestion. We collected the first sample 6–7 hours after dosing; for the male we collected another sample 24 hours post-dose (i.e., we attempted but were unable to find a vein within a few minutes to collect blood from the female at this time point), and we collected another post-dose sample 7 days after DLW ingestion for both individuals. Serum was separated from whole blood via centrifugation and transferred to a clean polypropylene tube. Serum samples were kept frozen (-80° C) and shipped in an insulated container to Duke University for isotopic analysis.

### Isotope analysis

We filtered samples using carbon black and a 30 kilodalton centrifuge concentrator (Vivaspin®). Subsequently, we determined enrichments of ^2^H (i.e., deuterium; D) and ^18^O using integrated cavity off-axis spectroscopy (ABB®). We used the slope-intercept method to determine the dilution spaces (i.e., N_D_ and N_O_) and the depletion rates (k_D_ and k_O_) for ^2^H and ^18^O, respectively [17–19]. We ran all samples in triplicate and used the average isotope enrichment for subsequent calculations. We calculated dilution space, N (moles), from N_O_ and N_D_ using equation 2 in Speakman et al. [20] as: N = (N_O_/1.007 + N_D_/1.043)/2 and total body water (TBW) was calculated from isotope dilution as TBW = 0.01802*N.

The isotope dilution space ratio (N_D_/N_O_) was 1.043 for the female and 1.045 for the male. We calculated the rate of CO_2_ production (mol day^−1^) following equation 1 in Speakman et al. [20] as: rCO_2_ = [(N/2.078)*(1.007*k_O_− 1.043*k_D_)-(0.0246*N*1.05(1.007*k_O_− 1.043*k_D_))].

We used rCO_2_ to calculate daily total energy expenditure (TEE, kcal day^-1^) using the Weir equation [21]: TEE = 22.26 rCO_2_ (1.106 + 3.94/FQ). FQ is the food quotient which reflects the macronutrient proportions of the diet whereby ME intake attributed to different macronutrients is multiplied by coefficients reflecting the oxidation rates of protein, carbohydrates, and fats, respectively. We estimated the FQ for our study subjects using the following equation: FQ = [(ME_CP_ × 0.8) + (ME_Carb_ × 1.0) + (ME_CF_ × 0.7)]/ ME_Total_. CP refers to crude protein, Carb includes the sum of ME intake attributed to neutral detergent fiber (NDF) and total nonstructural carbohydrate (TNC), and CF refers to crude fat. Fat free mass (FFM) was calculated by dividing TBW by a hydration coefficient of 0.732. We calculated fat mass (FM) by subtracting FFM from total body mass.

### Food and macronutrient intake

With the help of Cleveland Metroparks Zoo Animal Care staff, we quantified food and macronutrient intake of our study subjects throughout the study period from June 2020 to July 2021. Because the study animals were housed and fed separately, it was possible to determine individual food and macronutrient intake (female: n = 270 days; male: n = 256 days). We first recorded the mass of each food item offered per day. After controlling for moisture lost overnight based on experimental trials, we then subtracted the mass of each food item remaining to quantify the mass of various food items consumed. We quantified the mass of leafy browse consumed by stripping all leaves remaining from woody branches and subtracting that value from the initial mass offered, controlling for moisture lost overnight. Daily macronutrient intake was quantified by multiplying the mass of each food item consumed by the nutrient composition of each food item. We used published nutrient composition values from Schmidt et al. [22] for the majority of food items (e.g., leafy greens, fruits, and vegetables) offered to our study subjects. Leafy browse samples (n = 25 samples) were shipped to Dairy One Forage Laboratory (Ithaca, NY, USA) for macronutrient analyses following standardized procedures for ash (AOAC Method 942.05), crude protein (CP: AOAC 990.03), neutral detergent fiber (NDF: [23]), and crude fat (CF: AOAC 954.02). Total nonstructural carbohydrate (TNC) was estimated via subtraction: TNC = [100 - (%Ash + % CP + % NDF + % CF)].

### Estimation of apparent digestibility

Over a two-week period in June-July 2021, we collected all fecal samples for each individual over three consecutive days, and then repeated the procedure a week later, resulting in six days of total fecal collection per individual. Fecal samples were collected each morning at 10:00 hours, just prior to the morning feeding time. Fecal collection occurred only on days in which an individual was housed in the indoor off-exhibit holding area. Wood shavings and other substrates were removed from the concrete floor of the enclosure prior to the trials to facilitate fecal collection. Fecal samples were initially stored in a freezer (-20°C) for no more than 48 hours and then lyophilized using a freeze-dryer (Labconco 700401000 FreeZone 4.5L; Kansas City, MO, USA) for 72 hours. Dried fecal samples were weighed and homogenized using a mortar and pestle. Fecal samples (n = 12 samples) were shipped to Dairy One Forage Laboratory (Ithaca, NY, USA) for macronutrient analyses using the same procedures used for diet items outlined above. We used the average daily macronutrient intake (in g) over the two-week period and average fecal macronutrient output (in g) from the six sampling days to estimate apparent digestibility values of macronutrients for each Matschie’s tree kangaroo. We calculated apparent dry matter digestibility (D_a_) of each nutrient (N) using the following equation: D_a_N (%) = [(N feed intake−N _fecal output)_ / N _feed intake_] × 100.

### Metabolizable energy intake

We estimated metabolizable energy (ME) intake attributed to each macronutrient by multiplying standard physiological fuel values for CP (4 kcal/g), TNC (4 kcal/g), NDF (3 kcal/g), and CF (9 kcal/g) by the mass of each macronutrient consumed and then by the digestibility coefficient for each macronutrient calculated per individual [24, 25]. We then summed ME intake from CP, TNC, NDF, and CF to estimate daily metabolizable energy intake. Our estimates of ME intake do not account for energy lost in urine or methane.

### Statistical analyses

**Fig 1:**
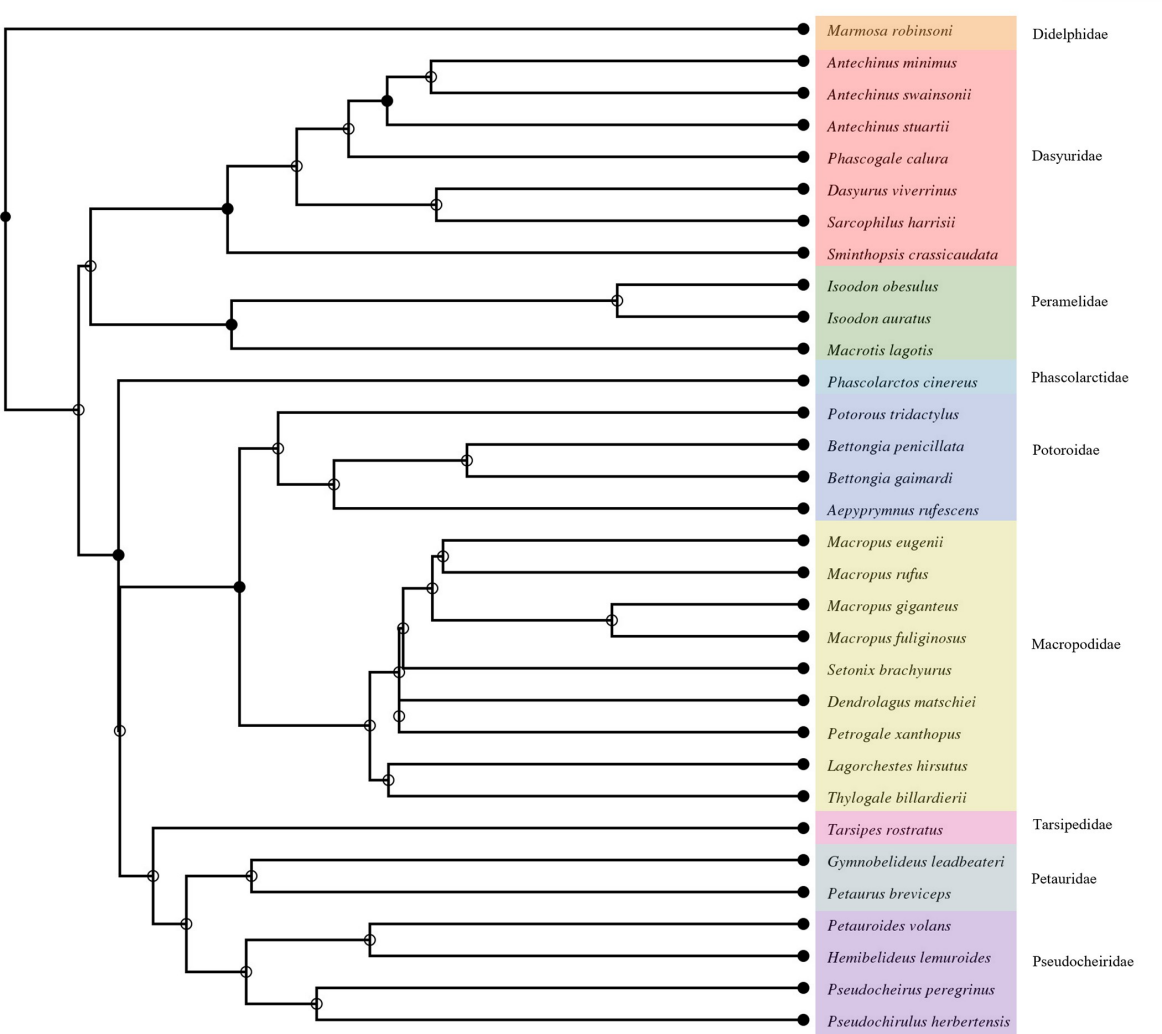
Phylogram of marsupial species used in the comparative TEE dataset. The phylogenetic structure was pruned from the TimeTree supertree (Kumar et al., 2017). Solid circles indicate nodes that map directly to the National Center for Biotechnology Information (NCBI) Taxonomy and the open circles indicate nodes that were created during the polytomy resolution process.

To compare Matschie’s tree kangaroo TEE values to those of other marsupials, we used published TEE values for 31 marsupial taxa (i.e., mean TEE per species) representing nine taxonomic families, including eight species of the Macropodidae [11, 26–29] (S1 Table). The phylogenetic structure of the analyzed species was pruned from the TimeTree supertree [30] (Fig 1).

We conducted all statistical analyses in R v.4.1.1. (http://www.R-project.org). To control for potential effects of phylogeny, we examined the relationship between log-transformed body mass and TEE using phylogenetic least-squares (PGLS) regression using the “pgls” function of the caper package [31]. We fitted models using a maximum-likelihood (ML) estimate for Pagel’s lambda [32]. We then compared Matschie’s tree kangaroo TEE from our study to expected values generated via PGLS regression from the overall marsupial dataset as well as the Macropodidae subset.

We examined seasonal differences in ME intake for each Matschie’s tree kangaroo using ANOVA. Because leafy browse represented a substantial proportion of the tree kangaroos’ overall diet (i.e., ~45–73% weight as-fed), seasons were defined based on when and where dietary leafy browse was acquired. We divided seasons into Cleveland’s early growing season (i.e., “summer”: May–July), Cleveland’s late growing season (i.e., “autumn”: August–October), Florida’s late growing season (i.e., “winter”: November–January), and Florida’s early spring growing season (i.e., “spring”: February–April). Post hoc pairwise comparisons were conducted using the emmeans R package [33]. Multiple pairwise comparisons were corrected using the false discovery rate method [34].

## Results

### TEE

**Fig 2:**
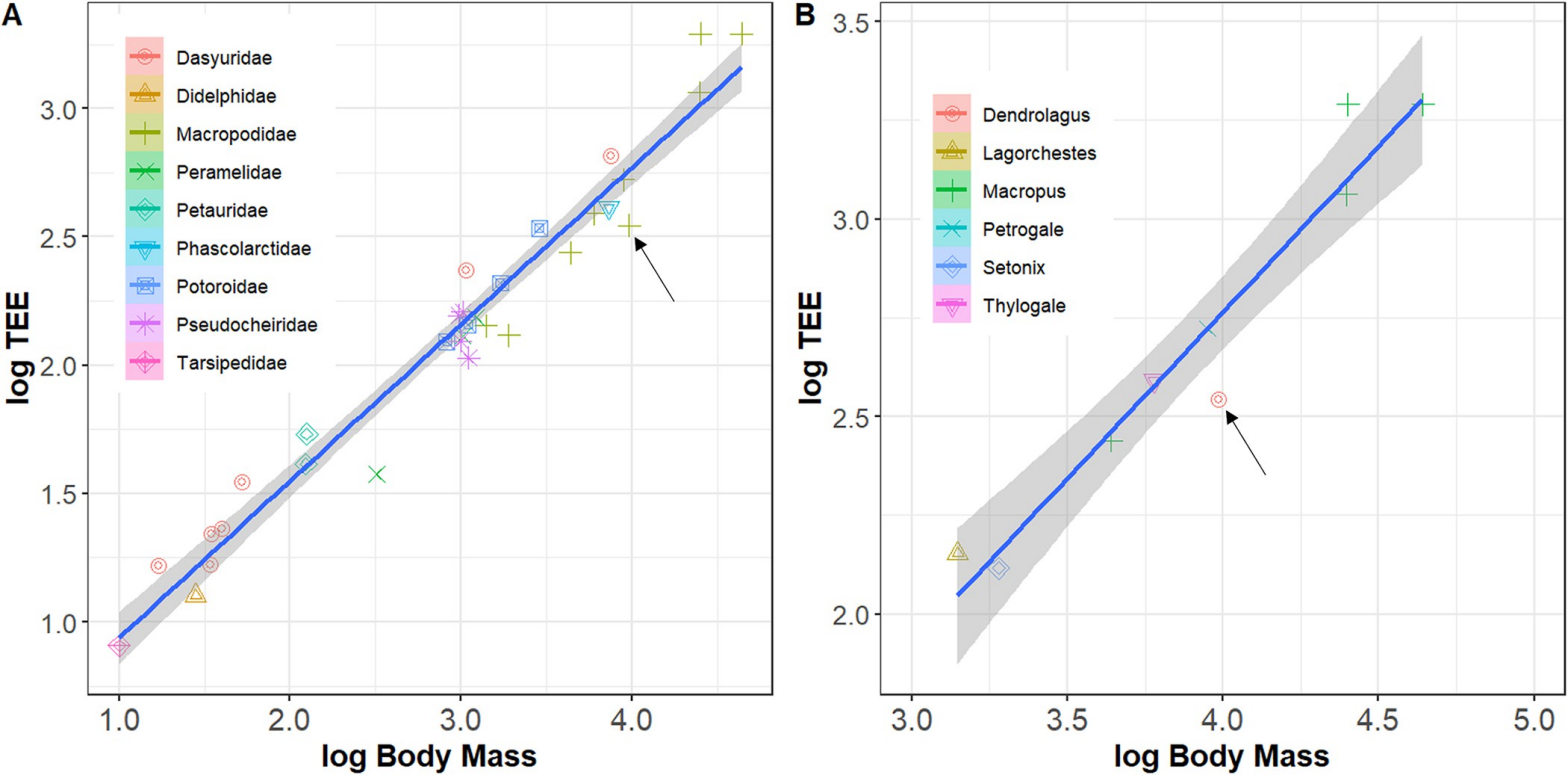
Comparison of TEE (kcal day^-1^) versus body mass (kg) for marsupials (A: n = 32 species organized by nine families) and Macropodidae (B: n = 9 species organized by six genera). Shaded regions indicate 95% confidence intervals for the linear regressions. Arrows indicate Matschie’s tree kangaroo (*Dendrolagus matschiei*) mean TEE value from this study. Published TEE values for 31 marsupial taxa taken from Hume [11], Munn et al. [26–28], and Sale et al. [29].

The mean TEE for the two Matschie’s tree kangaroos was 350 kcal day^-1^ (female: 288 kcal day ^-1^; male: 411 kcal day ^-1^). Table 1 lists key parameters used to estimate TEE values and also includes TEE values in kJ and scaled to body weight and metabolic body weight, respectively. The maximum likelihood value for Pagel’s lambda was 0 for both the larger comparative marsupial dataset and the Macropodidae subset, indicating no phylogenetic signal between body mass and TEE. Resulting TEE fell below the regression line for body mass vs. TEE. That is, Matschie’s tree kangaroo TEE represented 60.9% of the expected value (Fig 2A). Similarly, Matschie’s tree kangaroo TEE was below the expected value for body mass vs. TEE in the Macropodidae subset at 62.1% of the expected value (Fig 2B).

### Macronutrient intake and apparent digestibility

Table 2 shows the amounts of macronutrients in the ingested diet and fecal output over the two-week sampling period. Though not statistically compared, the proportions of macronutrients in the ingested diet and the fecal output are similar for the female and male Matschie’s tree kangaroo; however, apparent digestibility for all examined parameters is greater for the male.

**Table 2:** Macronutrient intake, fecal output, and apparent digestibility in zoo-housed Matschie’s tree kangaroos (*Dendrolagus matschiei*).

	Individual	
	Female	Male
**Ingested diet**		
DM (g)	147.9 ± 50.3	214.1 ± 57.8
CP (g)	24.9 ± 7.9	40.0 ± 9.8
NDF (g)	42.1 ± 16.5	56.5 ± 17.4
CF (g)	9.7 ± 4.1	12.1 ± 3.7
TNC (g)	61.0 ± 18.2	88.7 ± 26.8
**Feces**		
DM (g)	52.7 ± 13.0	42.6 ± 4.7
CP (g)	12.3 ± 2.7	10.1 ± 1.2
NDF (g)	20.2 ± 5.5	17.2 ± 2.0
CF (g)	3.5 ± 1.3	3.2 ± 0.4
TNC (g)	6.5 ± 2.9	2.5 ± 1.1
**Apparent Digestibility**		
DM (%)	64.4	80.1
CP (%)	50.8	74.7
NDF (%)	52.0	69.6
CF (%)	63.5	73.6
TNC (%)	89.3	97.2

Mean values reported with ± standard deviations. DM = dry matter; CP = crude protein; NDF = neutral detergent fiber; CF = crude fat; TNC = total nonstructural carbohydrates.

### ME intake

**Fig 3:**
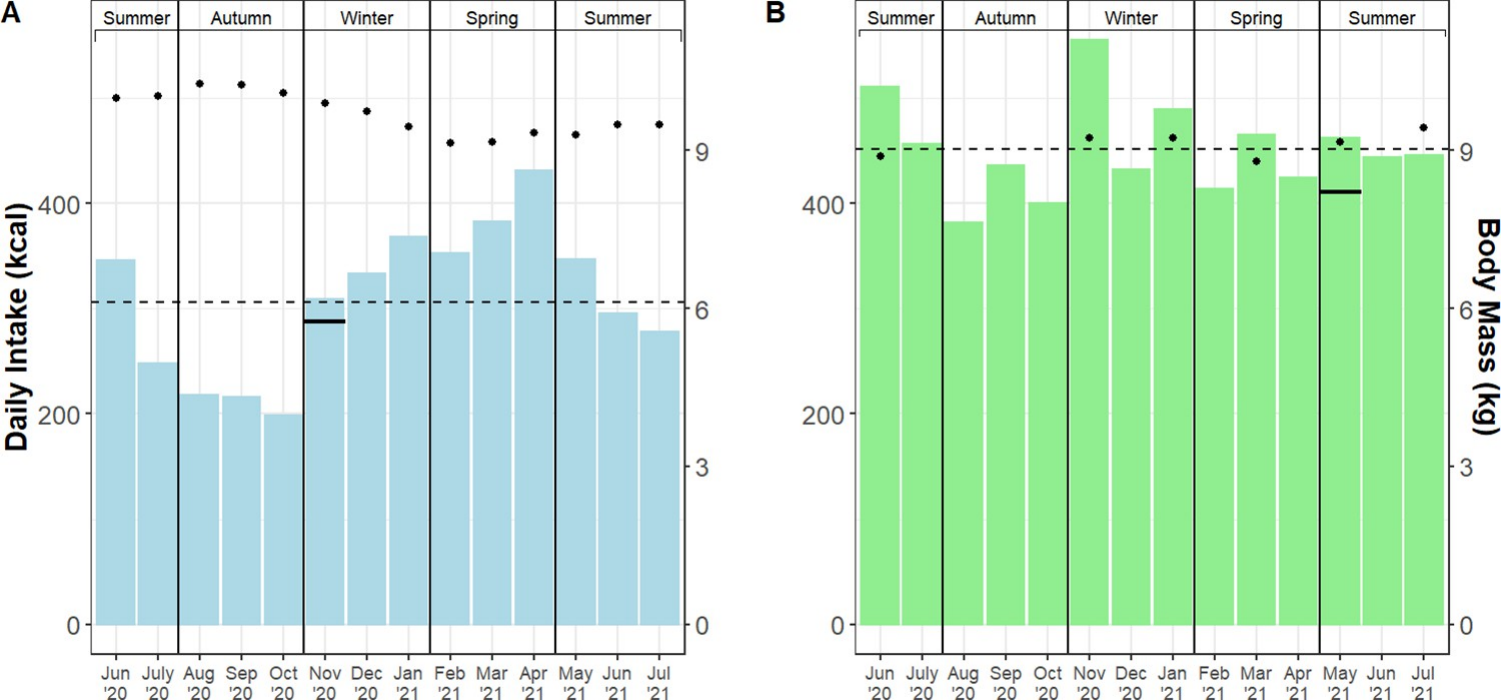
Daily ME intake (kcal day^-1^) and body mass (kg; secondary y-axis) by month for the female (A) and male (B) Matschie’s tree kangaroo (*Dendrolagus matschiei*). Solid horizontal line indicates TEE (kcal day^-1^) in the month it was quantified. Dotted horizontal line represents the mean ME intake over the course of the 14-month study. Black dots represent mean body mass value per month.

**Fig 4:**
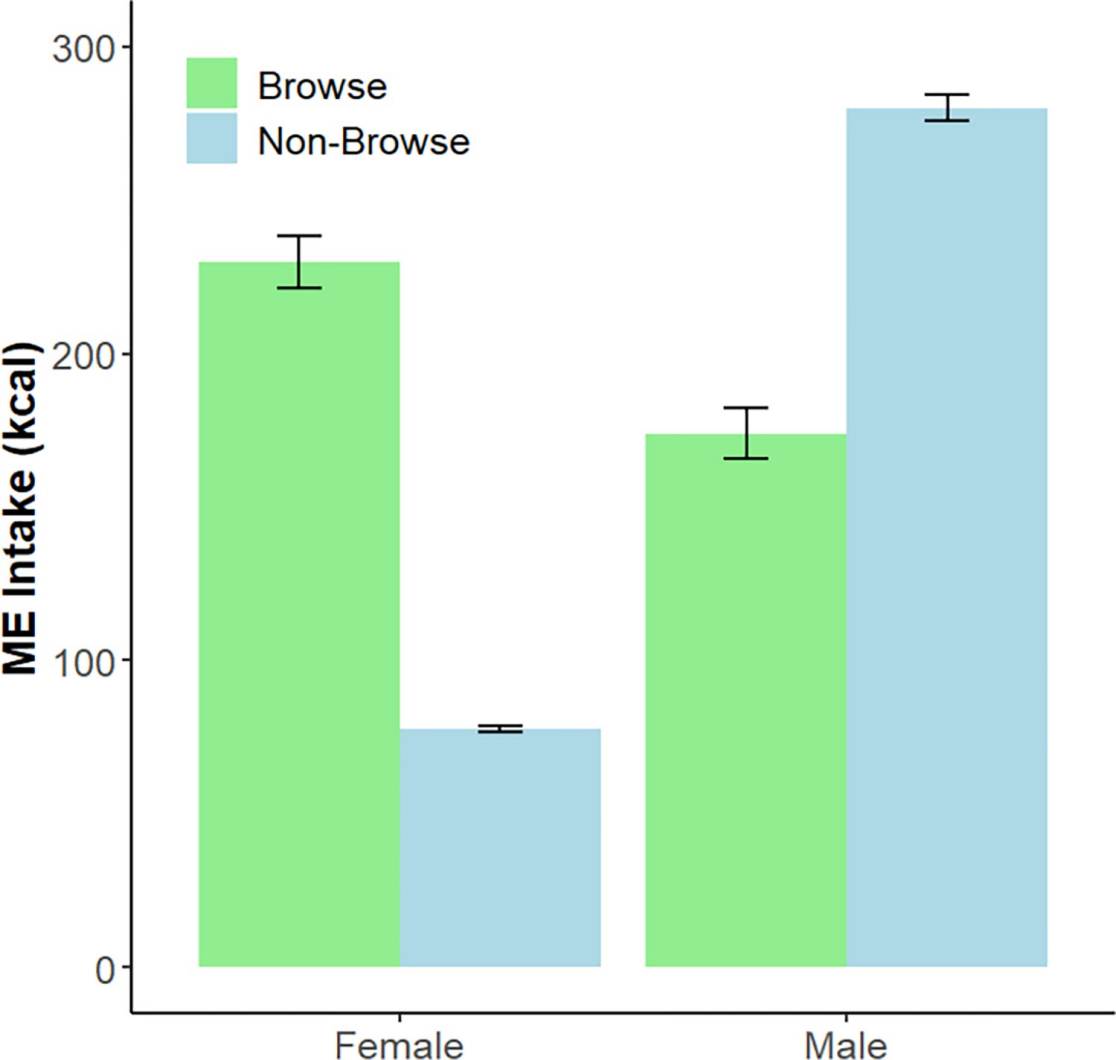
Daily ME intake (kcal day^-1^) attributed to browse and non-browse food items consumed by the female and male Matschie’s tree kangaroo, respectively, over the course of the 14-month study. Error bars indicate the standard error of mean values.

Over the fourteen-month study period, the mean calculated ME intakes for the female and male Matschie’s tree kangaroos averaged 307 ± 154 kcal day^-1^ and 454 ± 143 kcal day^-1^, respectively (Fig 3; S2 Table). These ME intake values were similar to the TEE values (female: 288 kcal day ^-1^; male: 411 kcal day ^-1^) reported above. There were significant seasonal differences in ME intake for the female (F_(3, 269)_ = 15.7; p < 0.001) and male (F_(3, 255)_ = 4.0; p = 0.008). These seasonal changes were largely driven by significantly reduced ME intake in the autumn season for both individuals (Table 3). Table 3 also lists ME intake values scaled to body weight and metabolic body weight, respectively. We quantified the daily ME intake attributed to leafy browse vs. non-browse items (i.e., vegetables, fruits, leafy greens, and dry goods). For the female, browse contributed 230 kcal day^-1^ (± 138 kcal) and non-browse contributed 77 kcal day^-1^ (± 14 kcal). For the male, browse contributed 174 kcal day^-1^ (± 132 kcal) and non-browse contributed 280 kcal day^-1^ (± 71 kcal) (Fig 4).

**Table 3:** Pairwise comparisons of mean seasonal ME intake in zoo housed Matschie’s tree kangaroos (*Dendrolagus matschiei*).

	Summer	Autumn	Winter	Spring
**Female**				
N days ME quantified	97	61	58	54
ME intake (kcal day^-1^)	302 ± 150^a^	212 ± 123^b^	338 ± 126^ac^	388 ± 166^c^
(kcal day^-1^ BW^-1^)	31 ± 15	22 ± 13	35 ± 13	40 ± 17
(kcal day^-1^ MBW^-1^)	55 ± 27	39 ± 22	62 ± 23	71 ± 30
(kJ day^-1^)	1264 ± 628	887 ± 515	1414 ± 527	1623 ± 695
(kJ day^-1^ BW^-1^)	130 ± 65	91 ± 53	146 ± 54	167 ± 72
(kJ day^-1^ MBW^-1^)	230 ± 114	161 ± 94	257 ± 96	295 ± 126
**Male**				
N days ME quantified	89	61	59	47
ME intake (kcal day^-1^)	466 ± 165^a^	407 ± 125^b^	490 ± 110^a^	435 ± 142^ab^
(kcal day^-1^ BW^-1^)	52 ± 18	45 ± 14	54 ± 12	48 ± 16
(kcal day^-1^ MBW^-1^)	90 ± 32	78 ± 24	94 ± 21	84 ± 27
(kJ day^-1^)	1950 ± 690	1703 ± 523	2050 ± 460	1820 ± 594
(kJ day^-1^ BW^-1^)	217 ± 77	189 ± 58	228 ± 51	202 ± 66
(kJ day^-1^ MBW^-1^)	375 ± 133	328 ± 101	395 ± 88	350 ± 114

Mean values reported with ± standard deviations. Shared superscripts within a row indicate that means were not significantly different. ME intake = metabolizable energy intake and is reported in multiple formats; BW = body weight in kg; MBW = metabolic body weight in kg and was calculated using the formula: MBW = BW^0.75^. Female and male ME intake values were not compared to each other.

## Discussion

We found that TEE of the two Matschie’s tree kangaroos tested here was remarkably low when compared to other marsupials and other members of the Macropodidae, specifically (i.e., mean TEE was roughly 60% of the predicted value based on body mass). Our findings are consistent with the fact that arboreal folivores generally have low BMR and/or TEE values across different mammalian taxa due to the energetic constraints of subsisting on nutritionally and energetically poor food resources [35–37]. While the feeding ecology of Matschie’s tree kangaroo has not been thoroughly studied, observations of this species at Yopno, Uruwa, and Som Conservation Area in Papua New Guinea suggest this species relies heavily on leaves from many species of trees, vines, and orchids [6]. Although this preliminary study only includes two individuals, measurements of TEE values are scarce for many taxa, and other studies on macropods suggest that adult conspecifics of the same sex, reproductive status, and body mass have remarkable similar TEE values [26, 27]. Analyses of additional Matschie’s tree kangaroos, particularly at different life stages, are needed, however, to allow more comprehensive conclusions to be drawn on the TEE of the *ex situ* population.

It is worth noting that these measurements for Matschie’s tree kangaroos lie even further beneath the marsupial regression line for body mass vs. TEE than does the more well-studied and notoriously slothful koala (*Phascolarctos cinereus*) which sleeps (or is inactive) for roughly 18–20 hours per day [38]. The activity of free-ranging and zoo-housed tree kangaroos has not been thoroughly investigated. While captive and zoo-housed animals may travel or locomote less than their free-ranging conspecifics [39, 40], TEE is often not significantly different when comparing captive vs. free-ranging conspecifics, including studies of giant pandas, red kangaroos, sheep, and multiple primate species [27, 41, 42]. We therefore expect free-ranging Matschie’s tree kangaroos to exhibit similar TEE values to the zoo-housed individuals in our study. Future studies of free-ranging tree kangaroos are required to test this prediction.

To effectively compare TEE to metabolizable energy intake, we needed to first quantify macronutrient digestibility. Macronutrient digestibility coefficients have not been determined for any tree kangaroo species, and digestibility coefficients are scarcely reported for members of the Macropodidae and folivorous marsupials. Our dry matter (DM) and NDF digestibility coefficient results are similar to those reported for koalas [43] and foregut fermenting folivorous primate species of similar body masses to Matschie’s tree kangaroos (reviewed in Hoshino et al. [44]); however, studies often do not report digestibility coefficients for CP, CF, or TNC. Studies have shown that diets richer in fiber are associated with lower digestibility of DM and NDF [44, 45]. This pattern is evident when examining the digestibility of the two Matschie’s tree kangaroos in our study. Though not statistically compared, the female consumed a diet composed of a higher percentage of leafy browse (i.e., 74.9% of ME intake from browse) resulting in an increased NDF intake compared to the male (i.e., 38.3% of ME intake from browse). This elevated NDF intake corresponded with lower CP and CF digestibility coefficients for the female. Similarly, our results for CP digestibility are consistent with the finding that a large portion of the CP in foliage (i.e., leafy browse) may not be accessible to folivores/herbivores because it is bound to tannins [46] and/or indigestible fiber [47]. Additionally, a substantial proportion of the CF may not be metabolizable because it takes the form of indigestible pigments, cutins, and waxes [48, 49]. Finally, we acknowledge the limitations of our dataset which includes digestibility coefficients for two individuals during one time period. Additional research is required to better understand how digestibility coefficients compare among individuals, seasons, and different diets offered to tree kangaroos at other facilities.

We also estimated daily ME intake over a fourteen-month period by combining detailed dietary intake records, macronutrient digestibility coefficients, and standard physiological fuel values. We found that mean ME intake over the study period was remarkably similar to TEE values for the female (i.e., ME intake: 307 kcal day^-1^; TEE: 288 kcal day^-1^) and male (i.e., ME intake: 454 kcal day^-1^; TEE: 411 kcal day^-1^), respectively. The congruence between TEE values and ME intake values during the months in which TEE values were quantified (i.e., Nov 2020 for the female and May 2021 for the male) suggests our individuals were in energy balance, at least during those periods of time. Interestingly, the female exhibited gradual weight loss throughout the winter season (i.e., body mass decreased from 10.3 kg in Aug 2020 to 9.1 kg in Feb 2021) despite exhibiting significantly greater ME intake in winter compared to the autumn season. It is important to note that this weight loss was desired by Animal Care staff for the female and helped her maintain an ideal body condition score [15]. It is possible that the observed weight loss during the winter season was a delayed response to reduced ME intake during the autumn season; however, further investigation is required to assess whether tree kangaroo TEE differs across seasons and the extent to which individuals adopt energy compensation strategies (e.g., adjusting resting metabolic rate and/or activity levels) to better match TEE to ME intake [50]. The male’s ME intake and body weight were more consistent throughout the study; however, we were unable to record his body mass as consistently as the female’s over the course of this investigation.

It is unclear why both individuals exhibited significantly reduced ME intake during the autumn season relative to some or all of the other seasons. The total amount of leafy browse offered per day remained largely consistent throughout the study period, but the variety and diversity of browses differed among seasons. Notably, the variety of browses offered declined in the autumn season, with elm (*Ulmus* spp.), mulberry (*Morus* spp.), and willow *(Salix* spp.) representing the bulk of the diet offerings compared to the other seasons in which 6–13 browse species were regularly offered on a rotational basis. Dietary mixing or switching among different foliage/browse species has been argued to be a strategy to avoid excessive ingestion of plant secondary metabolites unique to a given plant species [51]. Reduced browse variety/choice has been associated with decreased food intake in studies of the folivorous common brushtail possum [13, 14]. Furthermore, as the autumn season represents the end of the growing season and the end of our local browse procurement period, it is possible that the palatability of the browse species also declined during this season. While we did not systematically compare the nutrient composition of different browse species in early vs. late growing seasons, other studies have shown that the nutrient composition of temperate browse species changes across seasons, often with elevated NDF concentrations in the late growing season/autumn months [52–54]. Reduced browse consumption during the autumn may be due, in part, to the fact that arboreal folivores often prefer foliage with lower fiber concentrations [55, 56]. Preliminary investigations of browse preferences in our study subjects support this notion.

Though we recognize the limitations of our study with just two individuals, we nonetheless argue that our results have significant ramifications for captive management of Matschie’s tree kangaroos. Based on the low TEE values and ME intake values reported here, we argue that many zoos are overfeeding (i.e., offering too much food and easily digestible energy to their Matschie’s tree kangaroos) and this likely contributes significantly to a high number of individuals in the captive population that are overweight or obese. Prior to implementing a major diet reformulation in 2018 with the goal of reducing the weight of the female tree kangaroo in this study (i.e., she weighed as much as 12.2 kg in 2018), our tree kangaroos were offered ~490 kcal per day, not accounting for leafy browse which was offered more sparingly at that time. Similarly, preliminary analyses of other Matschie’s tree kangaroo diets reveal that some institutions are offering up to ~1300 kcal per individual per day, not including any calculated energy intake from leafy browse. Some individual tree kangaroos appear to self-regulate their food intake and maintain healthy body weights, while other individuals routinely consume the majority of, or all, food offered (Carlyle-Askew pers. comm., 2021). We recommend closely monitoring dietary kcal (or kJ) offered paired with regularly recording individuals’ body weights and body condition scores to help prevent obesity. For individuals that are already overweight or obese, total kcal offered should be gradually reduced, most easily through replacement of highly digestible items with higher fiber, less digestible ingredients.

We also advocate for using the DLW method to quantify TEE in additional Matschie’s tree kangaroos, across all life stages, as well as other zoo-housed taxa [41, 42, 57, 58]. This approach may be especially useful for species that are prone to obesity. Both blood and urine can be used for the DLW method. Many zoo-housed animals can be or are already trained to provide these samples voluntarily, and for some individuals, urine may also be collected opportunistically. Minimally, three samples are required to quantify TEE, including one prior to the DLW dosing and two timepoints post-dose, though there is some debate on the importance of collecting additional time-points post-dose [19, 59, 60].

In addition to the major discrepancy in energy offered to vs. energy required by tree kangaroos, the nutrient composition of commonly offered zoo dietary items is drastically different than the nutrient composition of items consumed by free-ranging Matschie’s tree kangaroos [6, 22]. Leafy browses have a macronutrient profile that is much more similar to that of items consumed by free-ranging Matschie’s tree kangaroos (i.e., greater NDF concentrations and lower starch and sugar concentrations) [6, 8]. Fibrous carbohydrates require longer gut retention times for processing, may contribute to improved satiety, and may help limit overeating. While many zoos offer leafy browses to their tree kangaroos, few provide substantial quantities and varieties or consider browse a primary diet item [7]. We have demonstrated that the tree kangaroos in this study can be maintained at healthy body weights and body conditions on a browse-heavy diet, representing ~40–75% of their total ME intake. In addition to the potential nutritional and physiological benefits of leafy browse, browse helps elicit natural foraging behaviors and has been associated with increased time spent foraging [61–64] and decreased rates of inactivity [61, 65], aggression [63], and stereotypic or undesirable behavior in a variety of zoo-housed taxa [62, 63, 66–69]. We argue that offering zoo-housed Matschie’s tree kangaroos a diet comprised of a majority of leafy browse provides a nutrient profile more similar to the wild diet of this species, assists with lowering ME intake without compromising animal satiety, and likely promotes the expression of natural behaviors and activity levels.

In conclusion, this is the first study to examine TEE, macronutrient digestibility, and ME intake in Matschie’s tree kangaroo. Our findings contribute important insights into tree kangaroo energetics and physiology and provide practical ramifications for the management and care of zoo-housed Matschie’s tree kangaroos. Notably, TEE was found to be considerably lower than expected based on body mass among members of the Macropodidae as well as among taxa from a larger marsupial dataset. Mean ME intake values were remarkably similar to TEE values. Taken together, our findings show that we can maintain Matschie’s tree kangaroos at healthy body weights (~9.0–9.7 kg) and body conditions (i.e., 5/9 or “ideal” condition [15]) on a fiber-rich and browse-heavy diet of roughly 300–450 kcal day^-1^. We stress that quantifying TEE in additional individuals and among different life stages is required to better understand variation in TEE and energy requirements among tree kangaroos. Finally, we recommend closely monitoring food and energy offered vs. that consumed, paired with regularly recording individuals’ body weights and body condition scores to help prevent obesity and potential downstream reproductive issues in zoo-housed Matschie’s tree kangaroos.

## Supporting information

S1 Table Comparative marsupial daily TEE dataset. (XLSX)

S2 Table Daily macronutrient and ME intake for two zoo-housed Matschie’s tree kangaroos throughout the study period from June 2020 –July 2021. DM = dry matter; CP = crude protein; NDF = neutral detergent fiber; CF = crude fat; TNC = total nonstructural carbohydrates, ME = metabolizable energy.
(XLSX)
